# Surprising Diagnosis of Myxedema Crisis In-Patient Who Presented With Altered Sensorium

**DOI:** 10.7759/cureus.33743

**Published:** 2023-01-13

**Authors:** Gajanan N Umalkar, Gajanan Chavan, Charuta Gadkari, Mayur B Wanjari

**Affiliations:** 1 Emergency Medicine, Jawaharlal Nehru Medical College, Datta Meghe Institute of Higher Education & Research, Wardha, IND; 2 Research and Development, Jawaharlal Nehru Medical College, Datta Meghe Institute of Higher Education & Research, Wardha, IND

**Keywords:** fatigue, cold intolerance, constipation, weight gain, myxedema crisis, hypothyroidism

## Abstract

Hypothyroidism, a disorder of decreased thyroid hormone secretion diagnosed by increased thyroid stimulating hormone (TSH) and low free triiodothyronine (FT3) and free thyroxine (FT4) levels, is classified as primary and secondary hypothyroidism, depending on the pathology. Raised TSH levels are associated with primary hypothyroidism, while decreased levels of TSH are seen in secondary hypothyroidism. With the easy availability of diagnostic tests, hypothyroidism can be detected and managed early but can be life-threatening if not treated within time. Manifestations of hypothyroidism are dry skin, hoarseness of voice, weight gain, constipation, cold intolerance, fatigue, and lethargy; however, the clinical presentation can differ as per age and sex and person to person. Here, we present one such case, which was brought to the emergency room with a history of altered sensorium, hypotension, and swelling over the bilateral lower limbs and face, with a surprise diagnosis of myxedema crisis. The uniqueness of this case is the omnipresent availability of early diagnosis and treatment in this era. still got a female patient with altered sensorium who was diagnosed to be a myxedema crisis which was given a lesser thought in our provisional diagnosis.

## Introduction

Hypothyroidism is an endocrine disorder that occurs due to a deficiency of thyroid hormones [1]. Thyroid stimulating hormone (TSH) values above the normal range and free triiodothyronine (FT3) concentrations below the normal range are referred to as primary clinical hypothyroidism. TSH concentrations above the normal range and free Triiodothyronine (T3) concentrations within the normal range are the criteria for subclinical hypothyroidism, frequently viewed as a symptom of early thyroid failure. Hypothyroidism can have major harmful effects and death if left untreated [2]. An adverse manifestation is myxedema crisis, which can be seen if the patient is left untreated chronically. Myxedema crisis is rarely seen nowadays because of readily available diagnosis and treatment facilities compared to older days. It is a life-threatening complication of hypothyroidism associated with altered mental status, hypothermia, and decreased metabolism. It can develop due to longstanding undiagnosed hypothyroidism or can be exacerbated by factors such as infection, myocardial infarction, cold exposure, and surgery in patients with poorly controlled hypothyroidism [3].

## Case presentation

**Figure 1:**
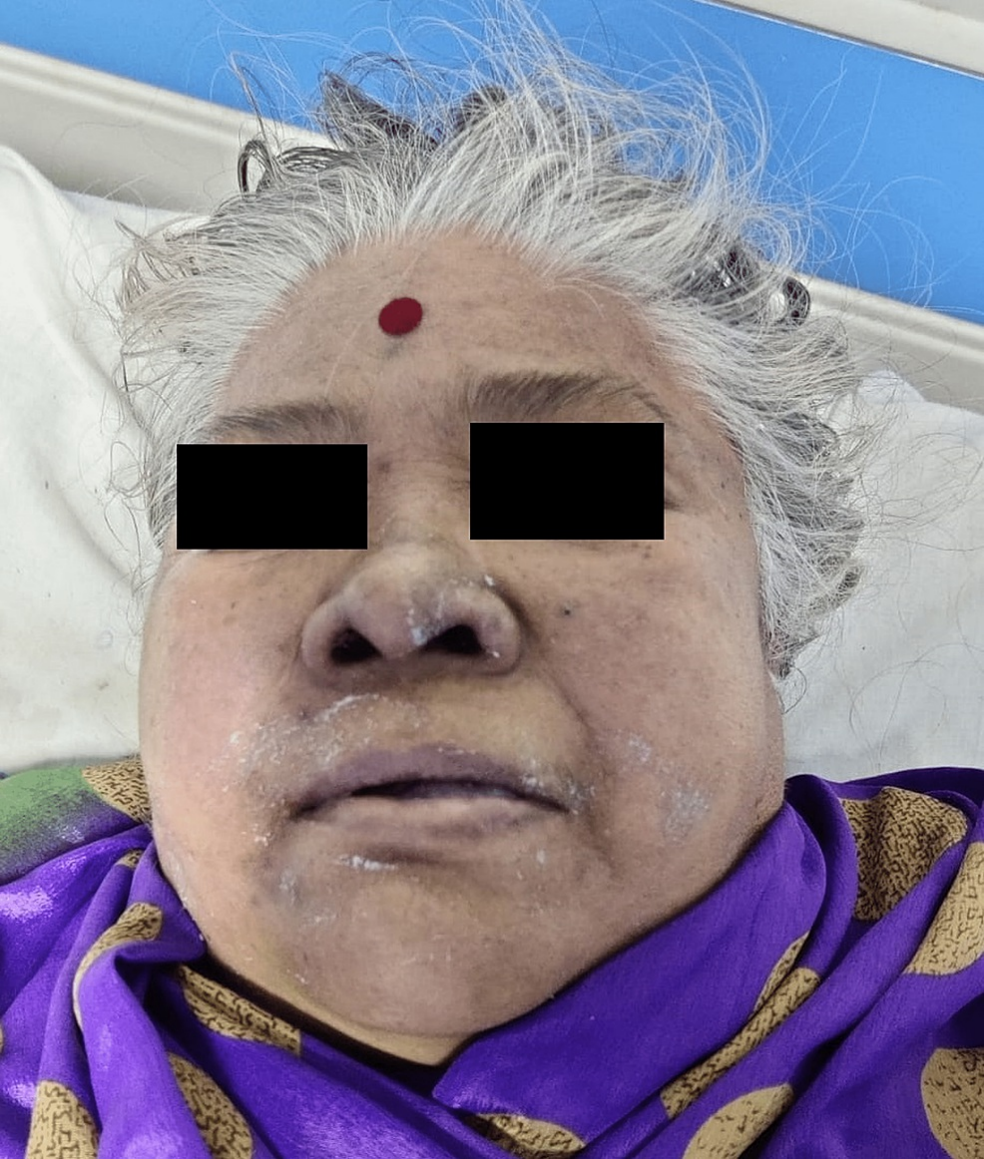
Shows Facial Puffiness

**Figure 2:**
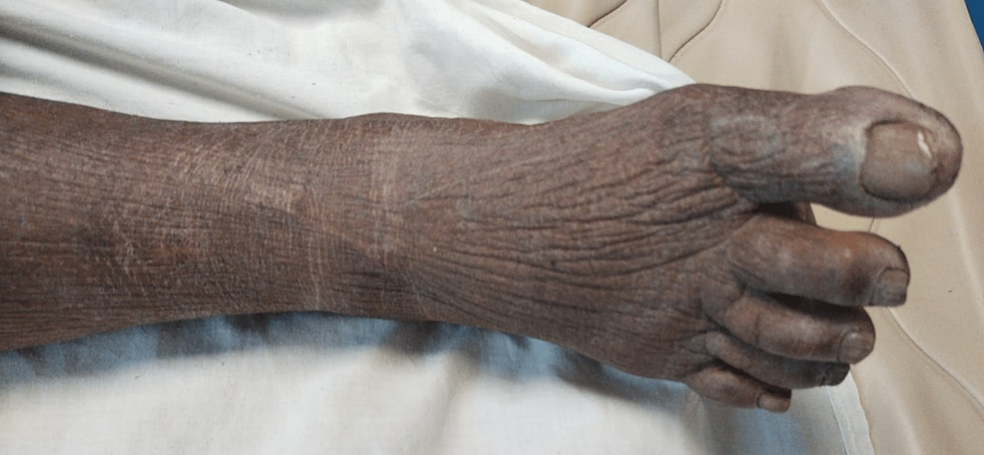
Shows Non-pitting Edema was Present with Dry, Coarse Skin Over the Right Lower Limb

A 70-year-old female was brought to the emergency medicine department by her son with a history of altered sensorium for three days. On presentation, her airway was patent with 70% saturation on room air, and air entry was absent on the right lower lobe on auscultation. Her pulse was at 75/minute and 70 mm hg of blood pressure with raised jugular venous pressure (JVP). She was drowsy but followed commands with Glasgow Coma Scale (GCS) E3, V5, and M6. Her blood sugar level was 120 mg/dl. Babinski's sign was negative, deep tendon reflexes were diminished, and neck stiffness and rigidity were absent on examination. Her extremities were cold, with a body temperature of 35.5 C. On local examination, the patient was pale with facial puffiness, as shown in (Figures 1, 2)

**Figure 3:**
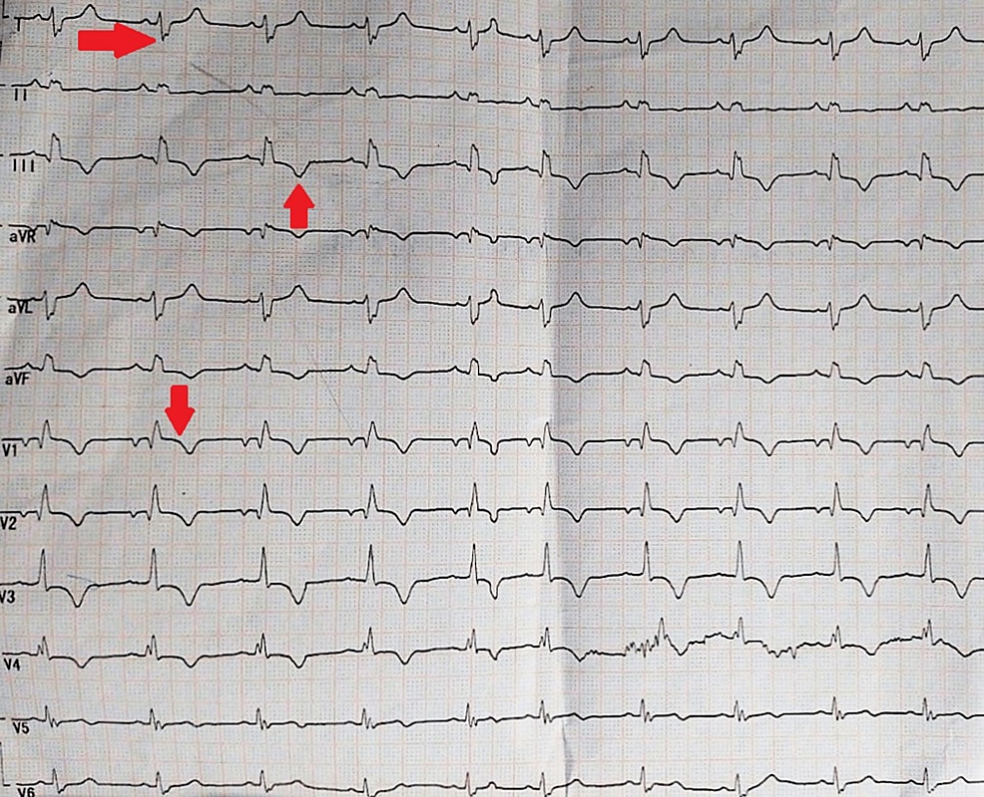
Shows Sinus Rhythm with Heart Rate at rate of 75/minute, S wave in lead I, Q wave in lead III, T inversion in lead III, Right axis deviation, Right bundle branch block, RV strain pattern in lead V1 and T inversion in lead avF

Her abdomen was distended, there was a dull note on percussion, and bowel sounds were sluggish on auscultation. Propped-up position oxygen was started via Hudson mask at a rate of 14 lit /minute, and 12 lead ECG was done as shown in (Figure 3). 

Arterial Blood Gas (ABG) was done, which was suggestive of uncompensated respiratory acidosis, which gets improved after management initiation as shown in (Table 1).

**Table 1:** Arterial Blood Gas Readings of Patient MICU: Medical Intensive Care Unit, ph: potential of hydrogen, pCO2: partial pressure of carbon dioxide, pO2: partial pressure of oxygen, HCo3: bicarbonate

Serial No	Blood gas parameters	On presentation to ER	After 12 hours of treatment in MICU
1	pH	7.169	7.25
2	pC02	66.7 mmHg	55 mmHg
3	PO2	40.6 mmHg	166 mmHg
4	HCO3 -	18.9 mmol/L	21.3 mmol/L

**Figure 4:**
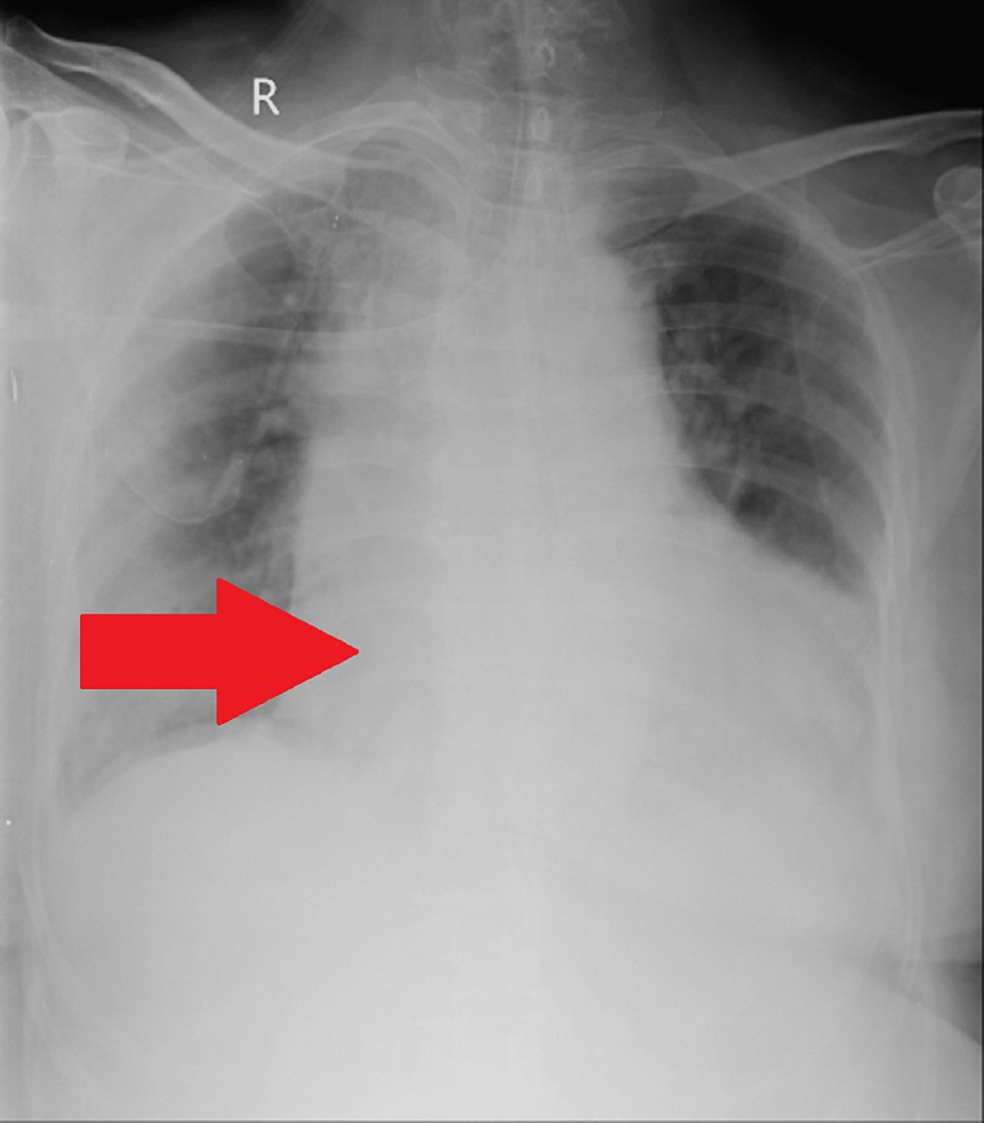
Chest X-ray Anterior-Posterior (AP) View - Demonstrate Massive Left Ventricular Hypertrophy. Widening of Mediastinum with Globular Shape is Consistent with Pericardial Effusion

There was a history of deep vein thrombosis of the left lower limb ten years back. The swelling persisted in the bilateral lower limb for ten years, and the patient was not on any treatment. She had no history of hypertension, diabetes mellitus, bronchial asthma, tuberculosis, hypothyroidism, seizures, or loss of consciousness. Blood samples were withdrawn for Complete blood count, liver function test, renal function test, D-DIMER, TSH, free T3, and free T4. Results are shown in Table 2. Figure 4 depicts the chest x-ray anterior-posterior (AP) view. CT brain plain was normal.

**Table 2:** Complete Blood Investigation of Patient

Investigation	Patient value	Normal value
Hemoglobin	11.1 gm %	12- 15 gm%
Mean corpuscular volume	80.4 fl	83 – 101 fl
Mean Corpuscular Hemoglobin	25.4 Pgm	27- 32 Pgm
Mean corpuscular hemoglobin concentration	31.6%	31.5- 34.5%
White blood count (WBC)	18,200 cu.mm	4000- 10,000 cu.mm
Platelet	3,12000	Lacs /cu.mm
Kidney Function Test (KFT)		
Urea	113 mg/dl	15- 36 mg/dl
Creatinine	4.0 mg /dl	0.52 – 1.04 mg /dl
Serum sodium	127 mmol/L	137- 145 mmol/L
Serum potassium	6.0 mmol/L	3.5- 5.1 mmol /L
Liver function tests (LFT)		
Serum glutamic pyruvic transaminase (SGPT)	841 U/L	< 35 U/L
Serum glutamic-oxaloacetic transaminase (SGOT)	1121 U/L	14- 36 U/L
D- DIMER	1708 ng/ml	<500 ng/ml
Creatine kinase	382 U/L	30 – 135 U/L
Serum Cortisol	26.5 pg/ml	4.6-22.7 pg/ml
Thyroid-stimulating hormone (TSH)	17.8 uIU/ML	0.465-4.68 uIU /ML
Triiodothyronine (FT3)	0.5 pg/ml	2.77-5.27 Pg/ml
Thyroxine (FT4)	0.4 ng /dl	0.78- 2.19 ng /dl

The patient was started on Inj Levothyroxine 20 mcg IV bolus and Inj Pantoprazole 40 MG IV. Inj unfractionated Heparin 5000 IU IV was given for pulmonary thromboembolism. Inj Piperacillin and Tazobactam 2.25 GM IV was given.The patient was shifted to the medical intensive care unit (MICU) for further management. After 12 hours, the patient was conscious and oriented to time, place, and person with GCS 15/15 and maintained a blood pressure (BP) of 100/60 mm Hg.

## Discussion

Hypothyroidism is an endocrine disorder with decreased thyroid hormone secretion. If untreated, it might worsen and lead to myxedema coma or severe decompensated hypothyroidism. The first case was reported from the St. Thomas Hospital in London in 1879. Low intracellular T3 secondary to hypothyroidism is the primary underlying pathology in the myxedema crisis, leading to hypothermia and suppression of cardiac activity [4]. 

Hypercapnia can result in altered consciousness because of respiratory failure, obesity, pleural effusions, macroglossia, and glottic edema [5]. Effusions and anasarca can develop due to altered vascular permeability. Water retention and hyponatremia occur secondary to reduced glomerular filtration rate [6]. Sinus bradycardia, low voltage complexes, bundle branch blocks, complete heart block, and nonspecific ST-T changes in electrocardiogram have been recorded in myxedema crisis [7]. Hypoglycemia can result from decreased gluconeogenesis, and precipitating factors like sepsis and concomitant adrenal insufficiency may contribute to hypoglycemia. 

Attention should be paid to signs of severe hypothyroidism like dry skin, a hoarse voice, hypothermia, delayed tendon reflexes, non-pitting edema, and surgical thyroidectomy scar [8]. Our patient was brought to the emergency room with a history of altered sensorium. Her blood sugar was normal. Blood samples for complete blood count, liver function test, and renal function test were sent in suspicion of sepsis, Liver/ Renal pathology, and metabolic encephalopathy, which were deranged as shown in Table 2. CT brain (plain) was done for intracranial pathology, which was normal.

Serum TSH was sent, given generalized edema. Until we got values, the patient was administered antibiotics, antacids, antiemetics, and 100 ml of normal saline. Serum TSH was surprisingly raised with decreased FT3 and FT4, which explained the reason for the altered sensorium, hypoventilation, hypothermia, hypotension, hyponatremia, cardiomegaly, and pleural effusion. Diagnosis of myxedema crisis was considered. A similar case was reported by Acharya et al. [9], where she was a known hypothyroid 72-year-old female [10]. Our patient was not diagnosed case of hypothyroidism.

Twelve lead ECG was suggestive of sinus rhythm with heart rate 75/minute, S wave in lead I, Q wave in lead III, T inversion in lead III, right axis deviation, Right bundle branch block, R.V. strain pattern which raised a possibility of pulmonary embolism. D-DIMER was sent, raised (1704 ng/ml), and 2D echo revealed she dilated the right atrium (RA) and right ventricle (RV). With the clinical presentation of dyspnea, hypotension, desaturation on room air, and history of one episode of deep vein thrombosis, the diagnosis of pulmonary embolism was confirmed. Well’s, the score used for suspected pulmonary embolism is calculated to be 7.5 in our patient with prior history of deep vein thrombosis DVT (1.5 points), bilateral edema on lower limbs (three points), and clinical presentation with hypoxia, hypotension, and raised JVP (three points). Tachycardia (scored at 1.5) was not seen, probably [10] due to hypothyroidism. The patient was managed with a stat dose of injection levothyroxine 20 mcg IV and an injection of unfractionated heparin 5000 IU. The patient was shifted to MICU and continued on the same management line. Upon follow-up to MICU after 12 hours, the patient was conscious, oriented to time and place, and breathing comfortably on five lit/min of oxygen.

## Conclusions

The emergency physician encounters many patients with decreased sensorium and altered sensorium routinely and is screened for acute pathologies; however, endocrine disorder should be considered even without typical symptoms because it can lead to severe complications and increased hospital stay.
